# Exploring the effects of telehealth on medical human resources supply: a qualitative case study in remote regions

**DOI:** 10.1186/1472-6963-7-6

**Published:** 2007-01-11

**Authors:** Marie-Pierre Gagnon, Julie Duplantie, Jean-Paul Fortin, Réjean Landry

**Affiliations:** 1 Evaluative Research Unit, Quebec University Hospital Centre, Quebec, Canada; 2 Department of Family Medicine, Laval University, Quebec, Canada; 3 Department of Social and Preventive Medicine, Laval University, Quebec, Canada; 4 Department of Management, Laval University, Quebec, Canada

## Abstract

**Background:**

The availability of medical human resource supply is a growing concern for rural and remote communities in many countries. In the last decade, various telehealth experiences in Canada have highlighted the potential impact of this technology on professional practice. The purpose of this study was to explore physicians' and managers' perceptions regarding the potential of telehealth to support recruitment and retention of physicians in remote and rural regions.

**Methods:**

A case study in Eastern Quebec was performed to explore this complex phenomenon. The analytical framework was based on two literature reviews and a Delphi study. Data were collected from semi-structured interviews with 41 physicians and 22 managers. Transcripts were produced and interview content was coded independently by two judges and validated by an expert panel.

**Results:**

Interviews have highlighted the potential impact of telehealth on several factors influencing the recruitment and retention of physicians in rural and remote regions. The potential effects of telehealth on physicians' choice of practice location could be seen at the professional, organizational, educational and individual levels. For instance, telehealth could improve work satisfaction by allowing a regional on-call duty system and a better follow-up of patients. However, there are also certain limits related to telehealth, such as the fear that it would eventually replace all continuing medical education activities and onsite specialists in remoteregions.

**Conclusion:**

Telehealth is likely to have an impact on several factors related to medical workforce supply in remote and rural regions. However, the expected benefits will materialize if and only if this technology is properly integrated into organizations as a support to professional practice.

## Background

The uneven distribution of medical human resources between regions constitutes a problem in many countries. Physician supply in several rural and remote regions and shortages in certain medical specialties are particularly problematic in Canada [1]. The recent National Physician Survey [2] shows that physician shortage limits access to health care, especially in rural and remote areas and small cities. This situation is of major concern for decision-makers as well as the general population [3,4]. In Canada, various measures of medical human resources planning have been implemented both at the federal and provincial levels. In the Province of Quebec, the Ministry of Health and Social Services is currently assessing the impact of different strategies to favor physicians' attraction and retention in remote and rural regions [5]. The training, planning and distribution of health care workforce is a top priority for health services research in Canada [6].

Historically, strategies to increase physician supply in rural and remote areas have mostly included financial incentives, such as premiums and bursaries, but also recruitment of rural students in medicine, specific training program in rural medicine, and promotion campaigns [1]. However, the impact of various recruitment and retention strategies is changing over time. Shifts in values and lifestyles, as well as the changing profile of the new generation of physicians – women now outnumber men in US and Canada's medical schools – call for innovative recruitment and retention strategies. Work satisfaction is thus becoming an important factor of retention among physicians [7].

Over the last decade, new information and communication technologies (ICT) – such as telehealth – have entered the sphere of medical practice. Different studies suggest that telehealth could have a positive impact on medical practice [8,9]. The Quebec Ministry of Health has also identified telehealth as an effective strategy contributing to improve accessibility of health services in remote regions [5]. However, could telehealth also be part of the strategy for influencing recruitment and retention of the new generation of physicians?

A research project was conducted to explore the potential of telehealth as a strategy to improve the practice of health care professionals in rural and remote regions of Eastern Quebec. One of the particularities of this project is that it involved decision-makers in its different phases in order to facilitate knowledge sharing and utilization. This article reports on a qualitative study that was conducted in four remote regions of Eastern Quebec to explore physicians and managers' perceptions regarding the potential of telehealth to support recruitment and retention of physicians in remote and rural regions. Approval from the ethics committee of the Quebec University Medical Centre was received for this research project.

## Methods

Studying the impact of telehealth on physician recruitment and retention in remote and rural regions represented a challenge given that this technology is still relatively new. Consequently, a case study was chosen as the appropriate methodological approach since it allows exploring a complex phenomenon in close relation to its context [10].

Several research methods were used to develop the analytical framework. First, two literature reviews were conducted: the first on the factors influencing physician recruitment and retention and the second on the effects of telehealth. These reviews allowed identifying which factors associated with physician recruitment and retention could be impacted by the implementation of telehealth. A series of hypotheses regarding the potential impact of telehealth on physician recruitment and retention was then generated. Second, a Delphi study was conducted by the research team to get a consensus from Canadian telehealth experts on the proposed hypotheses [11]. Finally, an analytical framework was developed to illustrate the hypothesized relationships between telehealth and recruitment and retention factors.

As shown in the analytical framework, seven categories of recruitment and retention factors were identified by the literature review: individual (e.g.: [12,13]), family (e.g.: [14]), community lifestyle (e.g.: [15]), professional (e.g.: [16,17]), organizational (e.g.: [18]), educational (e.g.: [19,20]) and economic (e.g.: [21]). The Delphi study and the literature reviews showed that telehealth is likely to have an impact on a set of individual, professional, organizational and educational factors related to the recruitment and retention of physicians in rural and remote regions.

In-depth semi-structured interviews were conducted individually and in small groups with 41 physicians (general practitioners and specialists) and 22 managers (hospital managers, recruitment specialists, and regional health managers) of Eastern Quebec. The characteristic of the respondent are presented in Table 1. Eastern Quebec was selected for the present study because several wide-scale telehealth projects have taken place there over the last decade [22-24]. Initial respondents for the interviews were selected through the network contact method [25]. Then a snowball sampling strategy [26] was used by asking initial respondents to refer us potential participants among their contacts. In order to accommodate respondents' busy schedule, interviews were conducted at their practice location, either individually or in small groups. Using principles of data saturation and information redundancy, data collection ended when additional interviews did not bring new information or opinion [27]. Interviews lasted 40 minutes on average. All interviews were tape-recorded with the consent of respondents and verbatim transcripts of the interviews were done.

**Table 1:** Characteristics of respondents

		**Physicians**		**Managers**		
		
**Variable**	**Total sample**	**General Practitioner**	**Specialist**	**Hospital Manager**	**Recruitment Manager**	**Health Region**
**Gender**						
Male (%)	37	5	19	8	2	3
Female (%)	26	4	13	3	3	3
Total (%)	63	9	32	11	5	6
**Clinical experience**						
Mean Standard deviation	15.7 ± 10.2	16.3 ± 8.9	15.5 ± 10.7	--	--	--
**Region**						
1 (493 to 1145 kms from the University Medical Center (UMC)	20	1	12	4	2	1
2 (279 to 2014 kms from the UMC)	14	3	6	2	2	1
3 (212 to 317 kms from the UMC)	15	3	8	3	--	2
4 (167 to 416 kms from the UMC)	14	2	6	2	1	2
**Utilization of telehealth**						
yes	43	2	24	9	2	6
no	17	6	8	--	3	--
Missing data	3	1	--	2	--	--

Two interview schemas were elaborated from the analytical framework: one for clinicians and one for managers. Interview schemas were validated with four extern collaborators of the research team who are experts on recruitment/retention and/or telehealth.

The interview schema for physicians was divided into three parts. The first part comprised questions about actual practice: work satisfaction; motivations for setting practice in the remote region; motivations for staying in the region; and potential factors that could make one leave the region. The second part covered perceptions on the effects of telehealth on clinical practice; as well as the benefits and limitations of telehealth use in one's practice. If respondents did not have access to telehealth, perceptions concerning its possible applications to their practice were gathered. The last part of the interview dealt with the possible effect of telehealth on recruitment and retention.

Managers from hospitals and health regions were also interviewed about the nature of their work and the strategies they were using to attract and keep medical workforce in their region. Managers were also asked questions dealing with their perception of telehealth benefits and limitations, and its effects on clinical practice, organization of care and recruitment/retention.

A qualitative iterative strategy was adopted for data analysis, based upon the method proposed by Huberman & Miles [28]. In a first step, all interview transcripts were read independently by two researchers to extract general impressions and preliminary classification categories based on the analytical framework. Classification categories were then compared and adjusted after a consensus discussion with the research team. In a second step, content of the interviews was codified according to this emerging classification. Data was then grouped into five broad categories: 1) recruitment factors; 2) retention factors; 3) work satisfaction factors; 4) potential impact of telehealth on recruitment and retention; and 5) potential impact of telehealth on work quality.

## Results

### Physician recruitment and retention factors

As shown in Table 2, participants' opinions regarding recruitment and retention factors have been classified into seven categories (individual, family, community lifestyle, professional, organizational, educational and economic), corresponding to those identified in the analytical framework. Professionals (occupational well-being) and organizational (critical mass) factors were the most frequently mentioned.

**Table 2:** Frequency of recruitment/retention factors mentioned by type of respondents

**Dimension**	Factors (frequency)*
Individual	• Native of the region (5Mn, 12Md)
	• Personality
	▪Challenge (5Mn, 7Md)
	▪Autonomy (2Mn, 3Md)
	▪Feeling of security (2Mn, 3Md)
	▪Polyvalent (1Mn, 4Md)
	▪Resourceful (2Mn, 2Md)
	▪Team work (1Mn, 1Md)
	▪Healthy (2Md)
	▪Easy to adapt (1Md)
Family	• Spouse influence (8Mn, 17Md)
	• Family integration (7Mn, 15Md)
Community lifestyle	• Quality of life/Social and cultural life (12Mn, 21Md)
	• Integration in the community (4Mn, 5Md)
Professional	• Occupational well-being (12Mn, 29Md)
	• Multiskill practice (9Mn, 18Md)
	• Technical support center (6Mn, 10Md)
	• Team reputation (7Mn, 7Md)
	• Specialist availability (4Mn, 8Md)
	• Integration to the team (4Mn, 7Md)
Organisational	• Critical mass (6Mn, 22Md)
	• Image of the center (4Mn, 3Md)
Educational	• Training in the region (7Mn, 6Md)
	• CME (3Mn, 7Md)
	• Effective education (3Md)
Economic	• Financial incitative (8Mn, 13Md)

* Number of managers (Mn) and/or physicians (Md) who mentioned the item.

Potential impact of telehealth on physician recruitment and retention factors When asked directly, interviewees generally agreed that telehealth could have a potential effect on their recruitment and retention. However, telehealth alone was not perceived as a direct recruitment/retention strategy, but rather as an element having an impact on important recruitment/retention factors. *"It *[telehealth] *can certainly help, it can't harm, that's sure. And it can be a part of an item of the basket that has a lot of other items included in the recipe for retention. One of the items (physician, region1)"*

Furthermore, interviews with physicians and managers showed that telehealth's impact on recruitment and retention in remote/rural regions would principally concern four of the categories identified in the analytical framework: professional, organizational, educational and individual factors

### Professional factors

#### Favour occupational well-being and decrease workload

Occupational well-being is the factor on which telehealth could have an impact that was mentioned most often. *"For it *[telelhealth] *to have an impact on retention, it is necessary that it has an impact on the quality of life or the quality of life at work" (physician, region 2)*. Telehealth is also perceived as a way to decrease workload, by allowing on-call duties to be done from home or shared on a regional basis. This allows the coverage of specialties such as radiology to be shared by specialists from several hospitals rather than those of a single hospital. *"Since at least six months, we have implemented a regional on-duty call system which covers the three hospitals of the area *[...]. *The four radiologists are ensuring coverage in turn, one week each." (physician, region3)*

#### Access to technology

One factor emerging from interviews and that was not mentioned in the literature is that telehealth could give access to the same technologies as other centers. This factor can decrease some of the frustration of being "low-tech" associated with rural/remote medical practice by providing remote access to high-tech equipment. *"It's a question of being able to practice without the frustration of not having the same technological means as the other centers. Being in region should not mean treating worse than elsewhere. To offer the patient to feel a bit like, how can I put it, he is injured because you work in remote region, you cannot use this technique *[since you don't have the technology].*" (physician, region 4)*

#### Professional support

Telehealth gives remote physicians the possibility to get support from their colleagues in other centers, to maintain contacts with their peers by giving access to team meetings for specialists, and to obtain a second opinion. This could reduce work pressure for remote physicians: *"Where there can be a need for telehealth is when you are alone and that you need a second opinion because there are decisions which are not easy to take. Me, I would not be able to work alone." (physician, region 3)*

Physicians mentioned that these benefits associated with telehealth decrease the feeling of isolation often experienced in rural/remote region and could attract others physicians. " [the project's goals] *was for example to make us take part in the meetings of Friday morning where we would all be connected, we would be there, we would attend the meetings, we would attend the reading club, I found that fantastic *[...] *Yes, I find that it's really fantastic because what we suffer the most in remote region is of professional isolation." (physician, region 1)*

### Organizational factors

#### Access to specialized human resources

Many physicians underscored the importance of having a critical mass of specialized human resources as a factor of recruitment/retention. In fact, it is difficult to recruit new physicians if the number of physicians working in the hospital is already low since potential recruits will fear huge workloads. Some interviewees mentioned that telehealth could be a temporary solution to this situation by facilitating regional or at home on-call duty systems. *" *[Telehealth] *offers support to the remaining teams, because with some share, it's quite important. There is a workload that you're able to take, but after a certain moment, you become dangerous. As you are professional, you don't want to become dangerous, therefore you will protect yourself. The only way you do this, *[...] *is by not taking it anymore, so, you leave."(physician, region 2)*

#### Stabilization of services

Telehealth could also contribute to decrease workload by a stabilization of services. For instance, an interviewee described the possibility of using telepathology to secure access to pathology services when the local pathologist is away, thus allowing the continuity of the surgery service. *"Let's take telepathology. In regions with small teams of specialists who will necessarily take holidays and will go away, if the links are functional and allow us to keep the service in place even if there is nobody on the spot, well, I think that yes, because in surgery, when there is no pathologist, it doesn't go very well." (manager, region 2)*. Another similar example is the use of teleradiology to maintain 24/7 emergency services in small hospitals.

#### Avoid patient transfer

Participants who used telehealth considered that it can have a benefit for patients by avoiding unnecessary transfers. This benefit gives physicians the possibility to answer patients' needs in the region and to avoid some frustration. "*We have a radiologist and when he's not there, we have to transfer our cases to the regional center for echography. The PACS *[Picture Archiving and Communication System] *goes relatively well because we have the PACS technology here, so we can take the pictures here. We send them to the regional centre by telemedicine. The on-call radiologist takes a look at it and returns the information back to us by fax or now by computer. That's good. Pediatrics here, it's only a 24 hours observation. If we need more, we have to transfer again to the regional centre. There are always such elements that are irritating. An irritant as much for the regional centre because they have to be there, we depend a bit on them, and as much for the professional aspect, I would say, because we *[remote physicians]*are tired of always having to do transfers. Then sometimes the relations become a little cold. And the emergency doctor becomes tired to make phone calls, phone calls and phone calls." (physician, region 3)*

#### By-pass recruitment

Despite its numerous benefits, telehealth was not perceived as a panacea to medical workforce shortage in remote regions neither by physicians nor managers. For instance, most respondents agreed that telehealth could represent a solution in some situations, but that it will never replace the presence of a physician on site. " [...] *So the telemedicine for me is not a way to by-pass an adequate recruitment strategy respecting critical mass" (physician, region 4)*

#### Signify shortage of specialists

Some managers feared that using telehealth in their hospital would mean that they lacked the critical mass of specialists and therefore, could hinder the recruitment of new physicians. *"But I am not sure that it's that way I will recruit my specialists. On the contrary: if I say to new recruits that I have telemedicine, in any case I think they will think that I've got a shortage of specialists. [...] *But for us to attract new recruits, I think it's necessary to be seen as rather autonomous" *(manager, region 4)*.

### Educational factors

#### Regional medical training

With respect to educational factors influencing physician recruitment and retention, receiving training in a remote or rural region was the most frequently mentioned by the respondents. Thus, telehealth could support the organization of joint training programs between several rural and remote locations. Such programs could also give exposure to the regions and their medical teams.*"With the joint educational programs, people know you more, which means new recruits!" (physician, region 4) *Moreover, telehealth could be used to provide training from experienced rural/remote physicians to medical students. *"Tele education could enable us to transmit our knowledge and to better adapt the training of students. That would make it possible to increase their qualification level." (physician, region 1)*

#### Access to continuing medical education

Respondents also acknowledged the importance of continuing medical education (CME) on physician recruitment and retention. Telehealth could improve access to CME by eliminating travel time since physicians could access CME from their home or office at the moment of their convenience. Also, telehealth allows equal access to CME activities for all professionals from a department. *"Often doctors won't have access to it *[training] *because when you cumulate the travel time required to attend a training like that one, you cannot allow it. Two people could not have gone either. You cannot empty a whole service here. So, it's certain that it's not possible anymore and not even thinkable to have training activities like these unless they're given by telehealth. » (manager, region 1)*

#### Knowledge update

When asked about the benefits of telehealth, physicians pointed out the access to updated knowledge that increases competence and professional development: *"We had felt the need for internists in remote region to have an update, to be able to invite a cardiologist once per year by telehealth who could talk to us about what is new in cardiology*. [...] *What have changed? 'Cause sometimes it changes quickly. Instead of learning it in the newspapers, they would have seen the cardiology, pneumology, infectiology, pathology, oncology... They would get an update on 10–12 topics per year using telehealth." (physician, region 1)*

However, some physicians mentioned their fear that tele-education would reduce the number of CME activities they can attend outside the region. For remote and rural physicians, attending CME activities is also important to create new contacts with peers, to learn about new practices, to transfer their knowledge, and for socialization. *"First of all, you go out... secondly, you meet fellow-members, which you won't do if you say that you will get all your training via telemedicine. Congresses are more convivial, more friendly, there are more exchanges..." (physician, region 3)*. This potential drawback of tele-education could have a negative impact on both recruitment and retention.

### Individual factors

#### Feeling of security

Physicians and managers pointed out that telehealth could facilitate access to the support of mentors and colleagues from academic medical centers. This support could make new physicians feel more secure in their work. *"Telehealth would make young doctors more secure since they could say to themselves "yet, I have a link with other centers and then if something happens, I can always ask for opinion, etc". Perhaps it would influence their decision to settle in the region." (physician, region 4)*

However, a manager mentioned a potential drawback in the case of a new physician who would lack the individual qualifications to practice in rural/remote region. *"Each region must recruit professionals who are made to work in this region. It would not be desirable to recruit an insecure person who agrees to work there because there is telehealth. Recruiting someone who is not made for the region only because of telehealth will only increase resources turnover" (manager, region 1)*

## Discussion

### Recruitment/retention

**Figure 1:**
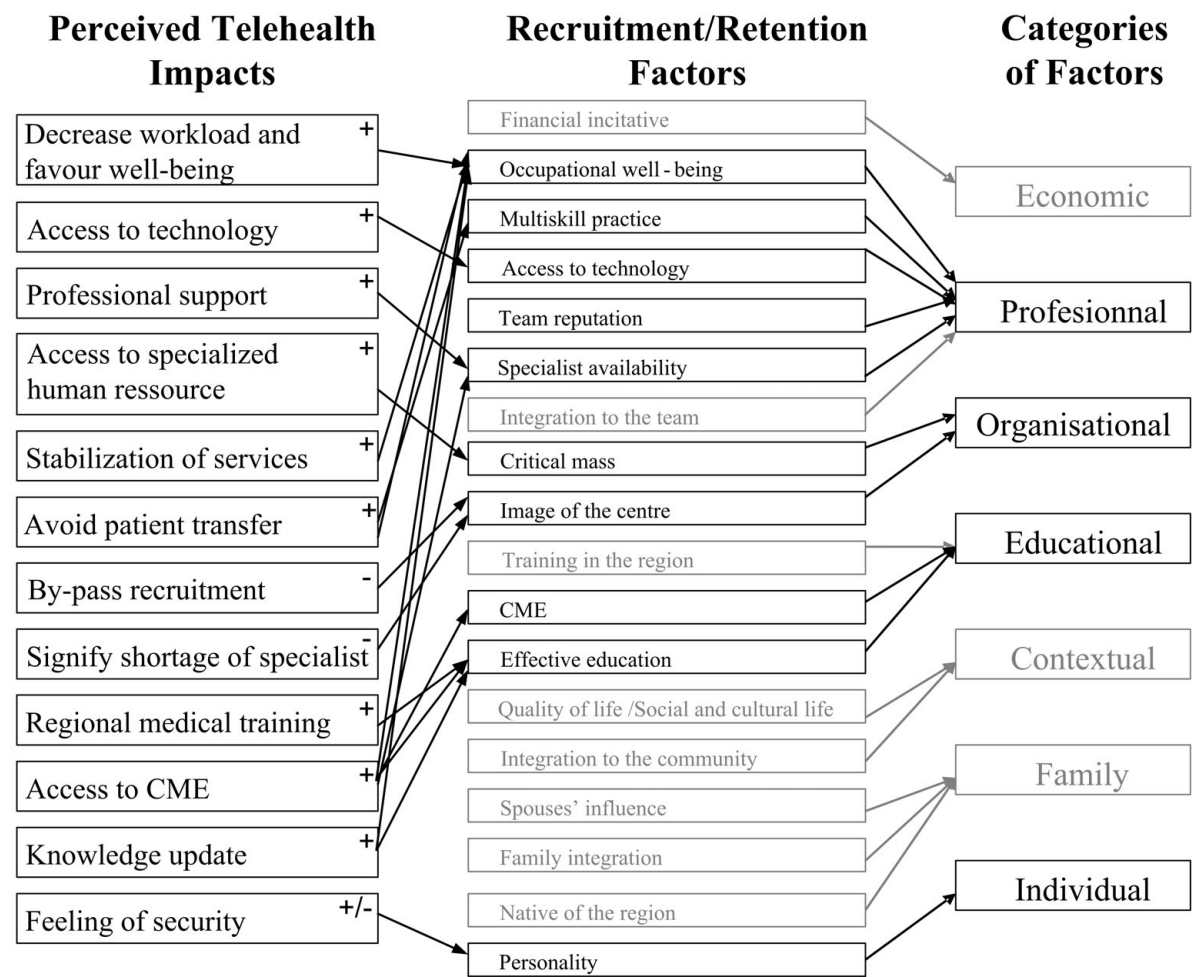
**Perceptions of respondents regarding telehealth impact on recruitment/retention factors**. Legend: + : perceived positive impact; – : perceived negative impact; +/- : perceived positive and negative impacts.

Seven broad categories of factors that influence the recruitment and retention of physicians in remote and rural regions were identified through the analytical framework. Interviews with physicians and hospital managers have highlighted the importance of many of these factors in the specific context of our study. We are thus confident that the present findings would apply to other jurisdictions with similar characteristics. Interviews conducted with physicians and managers indicate that telehealth could have an impact on four of the seven categories of factors described in the literature (professional, organizational, educational and individual) as shown in Figure 1.

More specifically, telehealth could have a positive impact on professional factors influencing physician recruitment and retention by increasing quality of life at work, supporting professional practice, and giving access to high technology.

Telehealth is also likely to have a positive impact on organizational factors that influence recruitment and retention by providing support to medical centers which do not have a critical mass of specialized physicians. Conversely, telehealth could have a negative impact by giving the image of a poorly equipped hospital.

With respect to educational factors, telehealth could have several positive effects. First, it can be used as a tool for providing adapted rural/remote medical training to students and residents. Second, tele-education increases access to CME which is an important factor of physician retention. However, one must make sure that tele-education will not replace all CME activities.

Finally, telehealth can have a positive impact on individual factors related to recruitment by increasing the feeling of security among new recruits, but this might have a negative impact on retention. Nevertheless, participants drew attention to the fact that such effects will be possible only if telehealth is better known and more used. Hence, the benefits reported here have to be taken as assumptions rather than as affirmations.

### Occupational well-being

**Figure 2:**
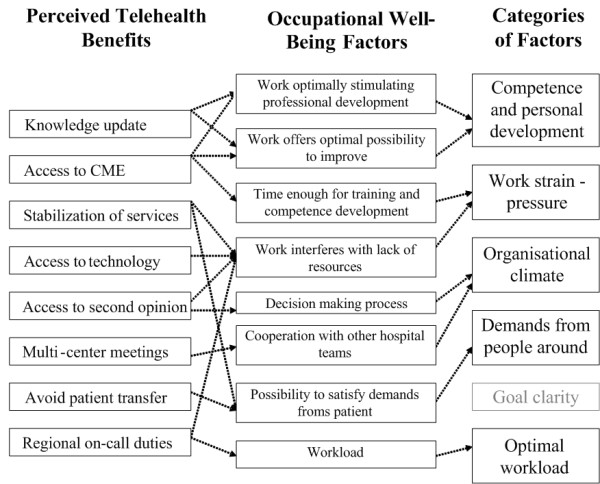
**Conceptualization of telehealth perceived benefits according to occupational well-being determinants**. *Based on Peterson and Arnetz's work quality model (1997).

The iterative approach used for analyzing qualitative data has allowed the development of a conceptual framework of the potential of telehealth to influence factors related to physician recruitment and retention. Study findings indicate that telehealth is likely to have a major impact on the concept of occupational well-being. This concept was used as a synonym to psychosocial work quality described in Petterson and Arnetz's model [29]. This model proposes six categories of factors that significantly impact psychosocial work quality: 1) Competence and personal development, 2) Work strain-pressure 3) Organisational climate 4) Demands from people around 5) Goal clarity 6) Optimal work load. Therefore, several of the telehealth benefits mentioned by respondents could have an impact on five of those factors, as shown in Figure 2. By facilitating access to CME and updated knowledge, telehealth could stimulate professional development and offer possibility to advance oneself. It could diminish work pressure by giving access to expertise and technologies that would not be easily available without telehealth. This technology could also improve the medical decision-making process by giving access to a second opinion. Moreover, telehealth could improve cooperation with colleagues form other hospitals by allowing multi-center meetings through videoconference. Together, these two factors are likely to improve the organizational climate. The stabilization of services and avoided transfers associated with the use of telehealth could also increase physicians' capacity to satisfy demands from their patients. Finally, telehealth could decrease physicians' workload by implementing on-call duty systems on a regional basis and from home.

## Conclusion

A number of observations can be drawn from this study. Obviously, telehealth could have an important impact on a set of professional, organizational, educational and individual factors related to medical workforce supply in remote and rural regions. However, the potential benefits of telehealth will materialize if and only if this technology is properly integrated into organizations and fits professional practices. Telehealth could improve occupational well-being, but to do so it has to be as easy and efficient as a phone call, as mentioned by many respondents. It can thus take several years before the effects described in this study can be measured. Nevertheless, this study has shown that even if telehealth can be seen as an asset for recruitment and retention of physicians, this technology alone cannot solve workforce shortages in remote regions. Finally, this comprehensive field study provides a framework to assess the potential influence of telehealth on medical human resources planning in the future. Thus, it could be interesting to estimate the weight of various recruitment and retention factors in the Quebec health system context. As telehealth technologies are becoming more present in the health care system, it would be important to follow the evolution of these various effects over time and in various settings through longitudinal studies.

## Competing interests

The author(s) declare they have no competing interests.

## Authors' contributions

MPG and JD prepared interview guides, conducted interviews, analysed interview transcripts and coded data. They proceeded to the literature review and wrote a first draft of the manuscript. JPF and RL were co-Principal Investigators on the project and gave feedback on research instruments and data analyses. All four authors revised and approved the last version of the manuscript.

## Pre-publication history

The pre-publication history for this paper can be accessed here:



## References

[B1] Task Force Two (2006). A Physician Human Resource Strategy for Canada: Final report.

[B2] College of Family Physicians of Canada, Canadian Medical Association, Royal College of Physician and Surgeons of Canada National Physician Survey (NPS): Workforce, satisfaction and demographic statistics concerning current and future physicians in Canada. http://www.nationalphysiciansurvey.ca/nps/home-e.asp.

[B3] Romanow RJ (2002). Building on Values: The Future of Health Care in Canada - Final Report.

[B4] Stoddart GL, Barer ML (1999). Will increasing medical school enrollment solve Canada's physician supply problems?. Cmaj.

[B5] Ministère de la Santé et des Services Sociaux (2001). Rapport annuel 2000-2001.

[B6] Dault N, Lomas J, Barer M (2004). Listening for direction II: National consultation on health services and policy issues for 2004-2007.

[B7] Matsumoto M, Okayama M, Kajii E (2004). Rural Doctors’ Satisfaction in Japan: a Nationwide Survey. Aust J Rural Health.

[B8] Gagnon MP, Fortin JP, Landry R (2005). Telehealth to support practice in remote regions: a survey among medical residents. Telemed J E Health.

[B9] Watanabe M, Jennett P, Watson M (1999). The effect of information technology on the physician workforce and health care in isolated communities: the Canadian picture. Journal of Telemedicine and Telecare.

[B10] Yin RH (1999). Enhancing the quality of case studies in health services research. Health Serv Res.

[B11] Duplantie J, Gagnon MP, Fortin JP, Landry R (2007). Telehealth and the recruitment/retention of physicians in rural and remote regions: a Delphi study. Can J Rural Med.

[B12] Cutchin MP (1997). Community and self: concepts for rural physician integration and retention. Soc Sci Med.

[B13] Levinson W, Lurie N (2004). When Most Doctors Are Women: What Lies Ahead?. Ann Intern Med.

[B14] Feeley TH (2003). Using the theory of reasonned action to model retention in rural primary care physicians. J Rural Health.

[B15] Szafran O, Crutcher RA, Chaytors RG (2001). Location of family medicine graduates' practices. What factors influence Albertans' choices?. Can Fam Physician.

[B16] Hankins RW, Guo L, Bentley LA (2002). Recruiting physicians and long-term viability: perspectives of physicians and practice managers. J Health Care Finance.

[B17] Humphreys JS, Jones MP, Jones JA, Mara PR (2002). Workeforce retention in rural and remote Australia: determining the factors that influence length of practice. Medical Journal of Australia.

[B18] Matsumoto M, Inoue K, Kajii E (2001). Rural practice evaluation: how do rural physicians evaluate their working conditions?. Aust J Rural Health.

[B19] Curran V, Rourke J (2004). The role of medical education in the recruitment and retention of rural physicians. Med Teach.

[B20] Rourke JT, Incitti F, Rourke LL, Kennard M (2005). Relationship between practice location of Ontario family physicians and their rural background or amount of rural medical education experience. Can J Rural Med.

[B21] Bilodeau H, Leduc N (2003). Recension des principaux facteurs d’attraction, d’installation et de maintien des médecins en régions éloignées. Cah Socio Démo Méd.

[B22] Bellavance M, Beland MJ, van Doesburg NH, Paquet M, Ducharme FM, Cloutier A (2004). Implanting telehealth network for paediatric cardiology: learning from the Quebec experience. Cardiol Young.

[B23] Berube J, Papillon MJ, Lavoie G, Durant P, Fortin JP (1995). Can a patient smart card improve decision making in a clinical setting?. Medinfo.

[B24] Fortin JP, Gagnon MP, Cloutier A, Labbé F (2003). Evaluation of a telemedicine demonstration project in the Magdalene Islands. J Telemed Telecare.

[B25] Morse J, Denzin NK, Lincoln YS (1994). Designing funded qualitative research. Handbook of qualitative research.

[B26] Johnson JC (1990). Selecting Ethnographic Informants. Anonymous Qualitative Research Methods Series No 22.

[B27] Morse J (1995). The significance of saturation. Qualitative Health Res.

[B28] Huberman AM, Miles MB (1994). Qualitative data analysis: An expanded sourcebook.

[B29] Petterson IL, Arnetz. BB (1997). Measuring psychosocial work quality and health: development of health care measures of measurement. J Occup Health Psychol.

